# Latin American Consensus: Children Born Small for Gestational Age

**DOI:** 10.1186/1471-2431-11-66

**Published:** 2011-07-19

**Authors:** Margaret CS Boguszewski, Veronica Mericq, Ignacio Bergada, Durval Damiani, Alicia Belgorosky, Peter Gunczler, Teresa Ortiz, Mauricio Llano, Horacio M Domené, Raúl Calzada-León, Armando Blanco, Margarita Barrientos, Patricio Procel, Roberto Lanes, Orlando Jaramillo

**Affiliations:** 1 Department of Pediatrics, Hospital de Clínicas, Federal University of Paraná, Curitiba, Brazil; 2 Institute of Maternal and Child Research, Faculty of Medicine, University of Chile, Santiago; 3 División de Endocrinología, Hospital de Niños "Ricardo Gutiérrez," Buenos Aires, Argentina; 4 Pediatric Endocrinology Unit, Instituto da Criança, São Paulo University Medical School, Brazil; 5 Hospital de Pediatria Garrahan, Buenos Aires, Argentina; 6 Unidad de Endocrinología Pediátrica, Hospital de Clínicas Caracas, Caracas, Venezuela; 7 Universidad Militar Nueva Granada Hospital Militar Central, Bogotá, Colombia; 8 Department of Pediatrics, Universidad del Bosque, Bogotá, Colombia; 9 Centro de Investigaciones Endocrinológicas (CEDIE-CONICET), Hospital de Niños "Ricardo Gutiérrez," Buenos Aires, Argentina; 10 Endocrinology Services, Instituto Nacional de Pediatría, México City, México; 11 Hospital Angeles Lomas, Ciudad de México, México; 12 Hospital Para el Niño Poblano, Puebla, México; 13 Instituto Ecuatoriano de Endocrinología y Metabolismo, IEMYR, Quito, Ecuador; 14 Unidad de Endocrinología Pediátrica, Hospital de Clínicas Caracas, Caracas, Venezuela; 15 Servicio de Endocrinología, Hospital Nacional de Niños "Dr. Carlos Sáenz Herrera," San José, Costa Rica

## Abstract

**Background:**

Children born small for gestational age (SGA) experience higher rates of morbidity and mortality than those born appropriate for gestational age. In Latin America, identification and optimal management of children born SGA is a critical issue. Leading experts in pediatric endocrinology throughout Latin America established working groups in order to discuss key challenges regarding the evaluation and management of children born SGA and ultimately develop a consensus statement.

**Discussion:**

SGA is defined as a birth weight and/or birth length greater than 2 standard deviations (SD) below the population reference mean for gestational age. SGA refers to body size and implies length-weight reference data in a geographical population whose ethnicity is known and specific to this group. Ideally, each country/region within Latin America should establish its own standards and make relevant updates. SGA children should be evaluated with standardized measures by trained personnel every 3 months during year 1 and every 6 months during year 2. Those without catch-up growth within the first 6 months of life need further evaluation, as do children whose weight is ≤ -2 SD at age 2 years. Growth hormone treatment can begin in SGA children > 2 years with short stature (< -2.0 SD) and a growth velocity < 25th percentile for their age, and should continue until final height (a growth velocity below 2 cm/year or a bone age of > 14 years for girls and > 16 years for boys) is reached. Blood glucose, thyroid function, HbA1c, and insulin-like growth factor-1 (IGF-1) should be monitored once a year. Monitoring insulin changes from baseline and surrogates of insulin sensitivity is essential. Reduced fetal growth followed by excessive postnatal catch-up in height, and particularly in weight, should be closely monitored. In both sexes, gonadal function should be monitored especially during puberty.

**Summary:**

Children born SGA should be carefully followed by a multidisciplinary group that includes perinatologists, pediatricians, nutritionists, and pediatric endocrinologists since 10% to 15% will continue to have weight and height deficiency through development and may benefit from growth hormone treatment. Standards/guidelines should be developed on a country/region basis throughout Latin America.

## Background

Low birth weight (< 2500 g) is prevalent in many countries and poses a significant public health problem contributing to a variety of short- and long-term negative effects. While about half of low-birth-weight infants in the industrialized world are born preterm (< 37 weeks' gestation), most of these infants are born full term in the rest of the world [1]. The United Nations Children's Fund State of the World's Children Report, 2003 [1,2], noted a 14% overall world prevalence of low birth weight, with the highest prevalence of low birth weight in South Asia (26%), with rates of 14% in developing countries, 9% in Latin America and the Caribbean (Table 1). About two-thirds of births in parts of Asia, Africa, and Latin America go unreported because many deliveries occur in small clinics or private homes [1], hence, it is logical to assume that the number of low-weight births is also underreported.

**Table 1:** Prevalence of low birth weight worldwide by region

Regions	% of infants with low birth weight 1995-2000
South Asia	26

Sub-Saharan Africa	12

Middle East & North Africa	11

**Latin America & Caribbean**	**9**

CEE/CIS and Baltic States	9

Least developed countries	18

World	14

Developing countries	14

Industrialized countries	7

Abbreviations: CEE, Central and Eastern Europe; CIS, Commonwealth of Independent States.

Source: United Nations Children's Fund 2003 State of the world's children report, 2003. New York: UNICEF, 2003. (2) Available at: http://www.unicef.org/sowc03/contents/index.html. Accessed September 8, 2010.

Higher rates of morbidity and mortality are more likely in children of low birth weight or born small for gestational age (SGA) than children born appropriate for gestational age (AGA) [3-5]. Hence, it is imperative to begin managing the health care of these children as early as possible to achieve optimal outcomes.

The development of the 2007 consensus statement on the management of children born SGA by the International Societies of Pediatric Endocrinology and the Growth Hormone Research Society [3] prompted leading experts to develop a similar consensus statement for Latin America. They held the first meeting in Lima, Peru, in October 2008, where three working groups discussed key issues to address within the consensus, specifically regarding the evaluation and management of children born SGA within Latin America. In March, 2009, the group met in Prague, Czech Republic, to further discuss and agree on the elements of a consensus document. Shortly thereafter, a consensus report was produced, which is presented here in final form. This paper presents a summary of the key issues discussed at the conferences regarding the clinical identification and optimal management of children born SGA.

## Discussion

### Definition of SGA

The definition of SGA is unclear. The World Health Organization (WHO) defines children born SGA or after intrauterine growth retardation (IUGR) as those with birth weight below the 10th percentile of the recommended gender-specific birth weight for referenced gestational age. Where gestational age is not available, the birth weight of < 2500 g should be considered low birth weight [6]. This definition is also used in obstetrics and neonatal guidelines due to the fact that these children represent groups with the highest neonatal morbidity and mortality [6-9]. However, neonates with either low birth weight or length--or both--for gestational age should be considered SGA.

For the purpose of this consensus, an SGA child is a child whose birth weight and/or birth length is at least 2 standard deviations (SD) below the mean for its gestational age [10].

Notably, we recommend that the term SGA not be used as a synonym for IUGR. The term IUGR refers to insufficient growth of the fetus and should only be used if at least 2 assessments of intrauterine growth are available and the fetus is not growing appropriately. SGA refers to body size--a low weight and/or length for a known gestational age--and is preferred in the absence of information about fetal growth.

### Epidemiology

Although many countries lack data on the actual incidence of children born SGA, there have been estimates ranging from 2.3% (< -2 SD) to 10% (< 10th percentile), depending on the definition used [11]. In Latin America, an evaluation between 1999 and 2004 at the Hospital Militar Central in Bogotá, Colombia, showed that 3.6% of 14,274 newborns were born SGA, defined as < 10th percentile weight and length (personal communication, Dr Teresa Ortiz). In Mexico, a review of 31,209 children born between 2000 and 2002 reported a 6% prevalence of SGA (≤ -2 SD weight) in the general population, ranging from 6.5% to 7.2% among salaried workers from Mexico City (n = 807) and from 3.7% to 6.9% in non-salaried farmers from small rural communities (n = 339) [12]. The discrepancy in SGA births among Latin American countries may also depend on which growth chart is being used, if it has been appropriately updated, and if it reflects the ethnicity mixture within a given country. In addition, the number of SGA births may also be impacted by the wide range of socioeconomic status and percentages of malnutrition that exist within Latin American countries.

### Initial identification

Considering the high morbidity and mortality rates in this population, prompt identification is critical. SGA newborns are 5 times more likely to die in the neonatal period and 4.7 times more likely to die in their first year of life [13]. Prematurity and low birth weight are significant causes of death in low- and middle-income countries [13,14]. SGA newborns are at a higher risk of developing hypertension and type 2 diabetes as adults [13,15,16]. They are also at higher risk of low IQ and presenting with short stature [13,15,16]. Children with low birth weight and subsequent early postnatal catch-up growth are at increased risk for obesity in childhood and disease in adulthood, including coronary disease, stroke, and diabetes mellitus--all of which are among WHO's top 10 causes of death worldwide [12,13,15,17,18].

Accurate determination of gestational age is essential for a diagnosis of SGA. The menstrual history of the mother and the use of ultrasound, usually in week 16 of gestation, increase the accuracy of the estimation. When this information is not available, physical examination of the newborn can be helpful (Ballard score [19]). Measurements of birth weight, length, and head circumference should be performed by trained personnel and follow appropriate, standardized procedures. The accuracy of newborn body measurements is paramount. Electronic scales for measuring weight and paper tapes for measuring head circumference are considered reliable methods [20-22]. Head circumference should be assessed at birth, as well as at first pediatric analysis during the first month of life in order to derive a more consistent measurement. Measurement of an infant's length can be less reliable [20,23], but its accuracy may be improved if the infant is measured by 2 people using a headboard [20,24]. The values should be compared with population-specific reference tables, allowing for classification as AGA or SGA, according to the chosen definition [25]. Country-specific reference charts for size at birth are preferred [26], but in some Latin American countries, these specific reference charts for size at birth are still unavailable. There is a critical need to develop reference charts for size at birth in each country, otherwise the definition of SGA could still be misleading in certain areas.

Growth references specific to Argentina have been used for more than 2 decades [27,28]. In 2009, Lejarraga *et al *published refinements to these charts by recalculating percentiles and lambda-mu-sigma (LMS) values from birth to maturity and incorporating updated WHO data from birth to age 2 years [29]. The Argentine growth charts are available at: http://www.garrahan.gov.ar/tdecrecimiento. When a national reference for preterm growth and size at birth is not available, the Babson and Benda growth charts updated in 2003 are recommended [20,30]. Children born prematurely (< 37 weeks gestational age) should be evaluated considering their special characteristics. Among them will be newborns SGA and AGA and appropriate references for preterm must be used [20]. However, it is important to recognize that defining SGA in preterm infants may be difficult since preterm reference charts do not typically include extremely preterm infants. Every country should make an effort to collect growth charts that represent a large number of premature infants of different gestational ages for more informed datasets.

### Causes of SGA

Many risk factors associated with low birth weight significantly overlap with risk factors associated with prematurely born infants. The characterization of an SGA infant should take into account the mother's height, weight, parity, age, ethnicity, and geographic location. Maternal malnutrition (ie, an insufficient weight increase during gestation), placental size and dysfunction, and the presence of maternal diseases should also be established [11]. Smoking, alcohol consumption, and drug use are preventable causes of IUGR; therefore, the mother's habits relative to these factors should be recorded. Fetal growth depends both on genetic factors and an optimal maternal-fetal health environment that allows the free flow of nutrients and oxygen, in addition to the integrity of growth factors IGF-1, IGF-2, and insulin synthesis and action. In addition, an excess of cortisol in fetal circulation produces a derangement in fetal growth.

### Growth and SGA

#### Follow-up

Most children born SGA recover from their weight and height deficiency. Term SGA newborns generally complete catch-up growth at about 2 years of age [31-33], whereas premature newborns may take longer to catch up than full-term newborns [34]. The recovery is completed when they reach their genetic potential as determined by parental height [33]. However, 10% to 15% of those born SGA will continue to have significantly short stature (height ≤-2 SD) during childhood and adult life [31,35,36].

Approximately 90% of healthy, full-term children born SGA will undergo catch-up growth during their first 2 years of life [31], which can occur as early as 12 weeks postnatal age [37]. As such, this consensus recommends that children born SGA should be evaluated every 3 months during the first year of life and every 6 months during the second year. Weight, length, and head circumference should be measured at all appointments. A child who does not show catch-up growth during the first 6 months of life should be evaluated further. The same recommendation is valid for a child whose weight is ≤ -2 SD at age 2 years. In these cases, common pediatric diseases, genetic disorders and hypothalamic and/or pituitary dysfunctions should be ruled out. SGA children who do not recover height generally have an adequate endogenous growth hormone (GH) secretion in response to pharmacological tests. However, they often have low serum IGF-1 levels and altered physiological GH secretion patterns [32,38].

Ideally, SGA implies length and weight reference data in a geographical population whose ethnicity is known. If local charts endorsed by local Pediatrics Associations are not available, from birth until age 5 years, the WHO growth charts could be used (http://www.who.int/childgrowth) [39]. The WHO charts combine data of a longitudinal follow-up from birth to 24 months and a cross-sectional survey of children aged 18 to 71 months; breastfed infants and young children from Brazil, Ghana, India, Norway, Oman, and USA were included. After age 5 years, the WHO Reference 2007--an update of the 1977 National Center for Health Statistics (NCHS)/WHO reference, using the original NCHS data set supplemented with data from the WHO child growth standards--is recommended http://www.who.int/growthref/en/[40].

### Criteria for initiation of growth hormone treatment

#### Age

GH treatment (somatropin [rDNA origin] for injection) was approved by the US Food and Drug Administration (FDA) in July 2001 for the long-term treatment of growth failure in children born SGA who did not have sufficient catch-up growth by age 2 years [10,41,42]. That is, children older than 2 years of age with either short stature (< -2 SD) and a birth weight < 2500 g at a gestational age ≥37 weeks [41], with birth weight or length < 3rd percentile for gestational age [41], or with ponderal weight index (100 × [weight in g] ÷ [length in cm]^3^) < -2 SD.

In Europe, GH treatment was approved by the Committee for Proprietary Medicinal Products (CPMP) in June 2003 for children born SGA (birth weight and length < -2 SD) with short stature (height < -2.5 standard deviation score [SDS] and parental adjusted height < -1.0 SDS) and who fail to show catch-up growth by age 4 years or older [43].

Argente and colleagues recently analyzed the outcome of very young SGA children, between ages 2-5 years, treated with GH [44]. They reported a greater increase in height velocity in the group younger than 4 years of age. Considering this finding, starting treatment shortly after age 2 years should be considered, particularly when growth velocity is below the 25th percentile for the child's age.

#### GH dosing recommendations

A clear dose-dependent increase in height velocity has been observed in children born SGA during the first years of treatment [10,45]. Optimal height is obtained with longer GH treatment before the start of puberty [46]. Furthermore, the height gain obtained with treatment during the prepubertal years is maintained until final height [46]. GH dosing recommendations for children born SGA differ in the US and Europe--the recommended dosage in the US is up to 0.48 mg/kg per week (68.5 μg/kg/day) divided into daily doses [41] and the corresponding dosage in Europe is 0.035 mg/kg/day continued until full height is reached [43]. We recommend an initial GH dose of 0.33 mg/kg/week (≈ 47 μg/kg/day or 0.15 IU/kg/day), with dose adjustments based on weight gain for those children with stature < -2 and > -3 SD. However, for those children with height < -3 SD, when rapid catch-up growth is desired, a higher dose might be indicated (0.48 mg/kg/week) without dose adjustment based on weight until they reach the regular dose of 0.33 mg/kg/week and IGF-1 levels within upper normal range. With this schedule a higher dose from the beginning of therapy will induce faster catch-up growth in those children who are more severely compromised.

#### Baseline assessment and follow-up during GH treatment

Baseline studies including hormonal (thyroid, IGF-1) and metabolic measurements (glucose, insulin, and lipid profile) are mandatory before initiation of GH treatment. Careful follow-up during GH treatment is recommended. The child should be evaluated every 3 to 6 months (physical examination and laboratory evaluation) by a physician experienced using GH to determine if dose adjustment is necessary [47]. Blood glucose, thyroid function, HbA1c, and IGF-1 should be monitored once a year except in cases exhibiting clear clinical evidence of insulin resistance or an HbA1C of 6% at the beginning of treatment. While it is necessary to measure IGF-1 levels once a year, twice a year is preferred. Monitoring changes from baseline insulin levels and surrogates of insulin sensitivity are also helpful in the follow-up of these children [48]. Notably, GH-associated adverse events are not more common in SGA than other conditions treated with GH [3]. Patients with a strong familial history of type 2 diabetes could be evaluated with the use of an oral glucose tolerance test (OGTT) at baseline and then as appropriate, contingent on 3- and 6-month laboratory values.

#### Criteria to stop GH treatment

Treatment with GH should continue if a positive growth response is observed within the first year of treatment (height velocity > +0.5 SDS) [3]. Treatment in adolescence should be stopped if the height velocity is below 2 cm/year and bone age is > 14 years for girls and > 16 years for boys, corresponding to closure of the epiphyseal growth plates [43].

#### Adverse events

Continuous GH therapy is not associated with serious adverse events in short children who are born SGA [49-51]. However, because of increased prevalence of metabolic disturbances and high blood pressure in adults born SGA [15,17,18,52-55], specific attention must be paid to glucose homeostasis and weight gain in short SGA children treated with GH. Previous studies have demonstrated that discontinuation of long-term GH treatment in SGA adolescents normalized insulin levels (both fasting and stimulated) after a significant increase during GH therapy [56,57].

GH treatment does not appear to be associated with an increased risk of malignancy [57]. It is recommended that IGF-1 concentrations should be monitored and GH dose should be reduced in children with a plasma IGF-1 above +2 SD [58,59]. In a long-term follow-up study of GH therapy in short children born SGA, increased IGF-1 levels were completely reversed after discontinuing GH [60].

### Puberty and SGA

#### Pubertal growth

Current data with regard to initiation, tempo, duration, and progression of puberty in children born SGA are limited and difficult to compare due to the differences in methodologies in the available literature (eg, SGA with or without catch-up, differences in the definition of SGA). Some studies show a normal but earlier pubertal development; however, others report a late pubertal start [61-63].

In human models, decreased pubertal growth has been observed [64]. In female models, a delayed growth of approximately 4 cm has been reported [65]. This finding suggests that there are gender-dimorphic susceptibilities for SGA postnatal pubertal changes [62,65]

With regard to the age of pubertal initiation, most studies report an age similar to that for children born AGA [62]; however, several researchers have shown either an earlier pubertal timing [61,65-67], or late tempo [68,69]. In an animal study analysis, investigators postulated that a higher level of exposure to insulin in postnatal life in those born SGA with accelerated weight gain may induce an earlier, exaggerated secretion of LH that leads to earlier pubertal timing [70].

Menarcheal age has also been reported as being earlier or within normal range [62,64,66,71-75]. Girls born SGA who had rapid weight gain in first months of infancy are more likely to have premature adrenarche. These girls can have earlier puberty and menarche than AGA girls with premature adrenarche [73,76].

Although bone age assessment is used to evaluate skeletal maturity, it has been suggested that use of bone age for final height prognosis is of limited value in children born SGA and thus should be used with caution [77].

SGA children without catch-up growth who are treated with GH have a normal pubertal timing and progression [68]. However, the use of GH near the beginning or late in puberty has not been shown to improve final height significantly [57,59].

#### Female gonadal function

Girls born SGA with catch-up growth present with lower insulin sensitivity, and there are data showing an increased incidence of adrenal and ovarian hyperandrogenism clinically evident as precocious pubarche [76,78,79]. However, these data were collected in a selected population from an endocrine clinic [76,78,79] and other studies have not confirmed these associations [80-84]. Thus far, there are insufficient data to confirm an altered ovarian function, fertility, or earlier menopause in these girls.

#### Metformin use

There is a preliminary report which suggests that the early use of metformin at a perimenarcheal age in low-birth-weight girls with a history of precocious pubarche prevents progression to polycystic ovary syndrome (PCOS); improves insulin sensitivity; and normalizes body composition, lipid profiles and GH secretion [85]. In a subsequent report in low-birth-weight girls with precocious pubarche by the same group, metformin treatment was associated with a less-adipose body composition; a 0.4-year delay in the clinical onset of puberty; a delay of at least 1 year in puberty-associated increase in circulating IGF-I; and a maintenance of height gain [86]. These findings remain to be confirmed by other studies and thus are still considered preliminary.

#### Male gonadal function

Little is known about the long-term effects of IUGR or being born SGA and their relationship to hypothalamic-pituitary-gonadal function in males [87]. Low birth weight has been associated with an increased frequency of hypospadia, cryptorchidism, and testicular cancer [87]. This cluster is known as testicular dysgenesis syndrome and a common fetal origin has been proposed for this association [87-91]. There are insufficient data about seminal quality and the relationship with catch-up growth or GH treatment in these individuals. On the other hand, some differences in the levels of pituitary and testicular hormones and in testicular volumes have been reported [92]. However, there is no consensus in this regard and no separation with individuals who had cryptorchidism [93]. Further studies to evaluate the impact of IUGR and SGA on the hypothalamic-pituitary-testicular axis are warranted.

### SGA and Metabolic Risks

A significantly increased risk of developing cardiovascular disease (hypertension and dyslipidemia) and type 2 diabetes during adulthood has been associated with low birth weight [52-54,94]. Three main hypotheses have been proposed to explain the association between low birth weight and increased metabolic risks. The fetal cortisol hypothesis postulated that maternal nutrient restriction may act to reprogram the development of the pituitary-adrenal axis, resulting in excess glucocorticoid exposure and adverse health outcomes in later life. In this hypothesis, placental 11 beta-hydroxysteroid dehydrogenase (11 beta-HSD) plays a key role by converting active cortisol to inactive cortisone. This enzyme guards the fetus from the growth-retarding effects of maternal glucocorticoids [95-97].

The second alternative hypothesis is the fetal insulin hypothesis which proposes that genetically determined insulin resistance results in impaired insulin-mediated growth in the fetus, as well as insulin resistance in adult life [98]. There is evidence to support this hypothesis in a minority of low-birth-weight cases. For example, monogenic diseases with impaired sensing of glucose, lowered insulin secretion or increased insulin resistance are associated with impaired fetal growth.

The most plausible explanation of this association, however, is the catch-up growth hypothesis. Children born SGA may present with a decreased insulin sensitivity early in life [15,99-101]. In the first study to evaluate insulin secretion and sensitivity in both SGA and AGA children from birth to age 1 year [99], investigators from the University of Chile, Santiago, reported that by age 1 year, the SGA children with catch-up weight gain (ie, weight gain SD score > 0.67) had higher fasting insulin levels and insulin resistance (insulin area under the curve during IV glucose) than AGA children. In a follow-up study of these subjects, the investigators reported that gains in weight SD scores continued to age 3 years in the children born SGA, and insulin resistance also progressed during this period [15]. At age 3 years, no differences in weight or BMI were seen between these SGA and AGA children, nor were there differences between the groups in first-phase insulin secretion. However, SGA children had a lower glucose disposition index (lower beta cell compensation) that persisted after postnatal weight gain. The SGA children also exhibited marked transition from lower pre-fed insulin and increased insulin sensitivity at birth to insulin resistance by age 3 years. The investigators noted this transition was related to rapid postnatal catch-up weight gain [15], which may be related to an increased tendency toward central fat deposition. They recommended long-term monitoring of glucose homeostasis in all SGA children regardless of postnatal catch-up growth. Thus, children born SGA should not be allowed to gain weight too rapidly or excessively in an effort circumvent the development of metabolic disturbances.

The pathophysiological mechanisms of insulin resistance are possibly secondary to a relative prolonged nutritional deficiency in the fetus, during which time the fetal metabolism constantly readjusts to slow growth with relative resistance to insulin, IGF-1, and GH. When this "adaptation" is inconsistent with postnatal nutrition, it may be associated with a rapid weight gain during infancy, and may result in considerable adaptation that develops a cluster of signs of metabolic syndrome [55,102] with insulin resistance as the key factor. This cluster increases the risk of experiencing comorbidities, such as obesity, diabetes, dyslipidemia, coronary disease, and hypertension, among others [103,104]. Indeed, the sum of risk factors (eg, rapid weight gain, family history, and ethnic group) all contribute to exacerbate the risk of metabolic syndrome in children born SGA. Notably, these children are not necessarily overweight or obese, but have a higher "adipose" body composition that contributes to the metabolic features [55]. In these cases, detection of clinical signs of insulin resistance, such as acanthosis nigricans, is of great importance. In addition, the periodic monitoring of blood pressure is vital in these children, especially if they are overweight or obese.

The efficacy of GH treatment in children born SGA has been confirmed by several series and by results in final height under continuous treatment with GH [3,44,46,51,57,105]. Nevertheless, safety in relation to glucose metabolism in GH-treated SGA children has been and continues to be an issue of particular importance, considering the known effect of GH on glucose metabolism. SGA children treated with GH experience a transient increase in serum insulin concentrations and variable degrees of compromised glucose tolerance [106]. These side effects, which are the result of a predictable response to the physiological actions of GH, have been previously reported [51,107-110]. However, no long-term effects have been observed on the prevalence of type 2 diabetes [107]. Furthermore, the effects on insulin resistance seem to be reversible [56,108,111,112].

In view of the fact that a high percentage of individuals with glucose intolerance have a fasting blood glucose level below 100 mg/dL, the OGTT is applicable in children and adolescents with risk factors such as obesity, family history, and hypertension. However, we do not recommend this be indicated as a general complementary test for children born SGA, rather, it should be individualized for children with rapid weight gain, acanthosis nigricans, dyslipidemia (high triglycerides), or a significant family history of metabolic syndrome [9].

Improvements in lipid profile and blood pressure associated with the metabolic syndrome in SGA children receiving GH treatment has also been reported [56]. Determining the concentrations of total cholesterol, HDL and LDL cholesterol, and especially plasma triglycerides are important. All of these values must be compared with appropriate standards for children of different ages.

### Implementation

Use of available population-, ethnic-, or country-specific reference data for birth weight and length, if available, is important for identification of SGA. However, accurate measurement of weight, length, and head circumference and, particularly, accurate gestational dating are more essential to a correct diagnosis of SGA [10]. In cases of suspected SGA, pediatricians should obtain ultrasonographic dating information, if performed during pregnancy. Pediatricians should also consider obtaining other pregnancy data, immediate perinatal/postnatal data, and early postnatal growth data, if available [10]. If possible, identifying the cause of SGA (eg, fetal factors [genetic, congenital anomalies], maternal factors, nutritional or substance abuse factors, uterine/placental factors, demographic factors, and multiple gestation), is important, as it may affect treatment. Children who do not experience catch-up growth should receive careful long-term physical and laboratory monitoring by an endocrinologist or pediatrician with endocrinology expertise. If necessary, they might be placed on GH therapy early [3].

Obstetricians/gynecologists should refer pregnancies with diagnosis of IUGR to a high-risk obstetric clinic with special care for certain diseases (eg, platelet dysfunction, hypertension) in order to reduce the severity of IUGR and the frequency of children born SGA. Although high-risk clinics are usually located in big cities, public health organizations should be aware of the needs of these patients and develop special networks for them. Neonatologists should be alert for the possible consequences of acute fetal distress that may occur during labor and/or delivery by caesarian section of an SGA infant, in addition to all the known complications such as respiratory distress, hypoglycemia, and hypokalemia.

A program for medical dissemination and training of multidisciplinary team members is an important next step. Training on the adequate management of SGA term and preterm newborns must be provided to pediatricians and neonatologists. Immediate, medium-, and long-term complications must be discussed, with an emphasis on growth problems and metabolic alterations that may develop. Pediatricians are encouraged to share their knowledge via multidisciplinary workshops. Furthermore, it would be important to provide written materials on the monitoring of adequate growth, the recovery phase, and follow-up in order to detect growth and metabolic alterations in these patients. Policies involving the primary care physician and pediatrician for detection and referral to a pediatric endocrinologist for the examination and treatment of growth and metabolic alterations in those born SGA should be developed.

## Summary

Most children born SGA recover from their weight and height deficiency. However, 10% to 15% of children born SGA will continue to have short stature. GH treatment benefits growth potential in short-stature children born SGA. In both sexes, gonadal function should be taken into account, especially during puberty. Reduced fetal growth followed by excessive postnatal catch-up in height, and particularly in weight, should be monitored. Excessive weight gain is most frequently associated with metabolic risk later in life. Finally, children born SGA should be carefully followed by a multidisciplinary group that includes perinatologists, pediatricians, nutritionists, and pediatric endocrinologists, in order to improve growth, glucose homeostasis, and gonadal function.

### Latin American SGA Consensus Guidelines Summary

• Local charts endorsed by local Pediatrics Associations are recommended. If local charts are not available, WHO growth charts may be used from birth until age 5 years, after which the WHO Reference 2007 is recommended.

• Most infants born SGA recover from their weight and height deficiency.

• GH treatment benefits growth potential in short-stature children born SGA.

• Gonadal function in both sexes should be taken into account, especially during puberty.

• Children born SGA should not be allowed to gain weight too rapidly or excessively in an effort to circumvent the development of metabolic disturbances.

• Children born SGA should be followed carefully by a multidisciplinary group.

• A program for medical dissemination and training of multidisciplinary team members is needed.

## Competing interests

Margaret CS Boguszewski is a member of the KIGS Strategic Advisory Board; she has no financial information to declare. Durval Damiani received less than $10,000 in honoraria from Pfizer Inc. This study was funded by Pfizer Inc.

All other authors declare that they have no competing interests.

## Authors' contributions

All the authors participated in the consensus meeting to discuss and vote on recommendations. MB, VM, AB, and IB helped draft the manuscript. All authors read and approved the final manuscript.

## Pre-publication history

The pre-publication history for this paper can be accessed here:

http://www.biomedcentral.com/1471-2431/11/66/prepub
